# Delayed reperfusion deficits after experimental stroke account for increased pathophysiology

**DOI:** 10.1038/jcbfm.2014.197

**Published:** 2014-11-19

**Authors:** Fiona E Burrows, Natasha Bray, Adam Denes, Stuart M Allan, Ingo Schiessl

**Affiliations:** 1 The University of Manchester, Faculty of Life Sciences, Manchester, UK; 2 Institute of Experimental Medicine, Budapest , Hungary

**Keywords:** inflammation, middle cerebral artery occlusion, optical imaging, reperfusion, stroke, spectroscopy

## Abstract

Cerebral blood flow and oxygenation in the first few hours after reperfusion following ischemic stroke are critical for therapeutic interventions but are not well understood. We investigate changes in oxyhemoglobin (HbO_2_) concentration in the cortex during and after ischemic stroke, using multispectral optical imaging in anesthetized mice, a remote filament to induce either 30 minute middle cerebral artery occlusion (MCAo), sham surgery or anesthesia alone. Immunohistochemistry establishes cortical injury and correlates the severity of damage with the change of oxygen perfusion. All groups were imaged for 6 hours after MCAo or sham surgery. Oxygenation maps were calculated using a pathlength scaling algorithm. The MCAo group shows a significant drop in HbO_2_ during occlusion and an initial increase after reperfusion. Over the subsequent 6 hours HbO_2_ concentrations decline to levels below those observed during stroke. Platelets, activated microglia, interleukin-1*α*, evidence of BBB breakdown and neuronal stress increase within the stroked hemisphere and correlate with the severity of the delayed reperfusion deficit but not with the ΔHbO_2_ during stroke. Despite initial restoration of HbO_2_ after 30 min MCAo there is a delayed compromise that coincides with inflammation and could be a target for improved stroke outcome after thrombolysis.

## Introduction

Stroke is one of the major causes of morbidity and mortality in the western world. Given the multiparametric response of the brain and immune system to an ischemic insult, there are few therapeutic interventions available.^1, 2^ In humans, the main cause of stroke is ischemic brain injury (85%), usually following from occlusion of the middle cerebral artery (MCA).^3, 4^ Ischemic stroke leads to hypoperfusion of brain tissue, inducing an energy-depleted state within the infarct core and triggering a number of acute pathophysiologic processes, altogether resulting in neuronal injury. Induction of hypoperfusion, i.e. the reduction in cerebral blood flow within the affected area, starves neurons of glucose^5^ and leads to the constriction of pericytes,^6^ the release of endogenous mitochondrial damage-associated molecular patterns,^7^ and proinflammatory cytokines.^8, 9^ To improve treatment of ischemic stroke, it is important to understand the underlying pathophysiologic changes that occur within the acute phase of cerebral ischemia.

Hypoperfusion that results in a deficit in the delivery of oxygen to cerebral tissue within the infarct core causes irreversible changes to neuronal integrity and vascular components that contribute further to the lack of reperfusion.^6, 10^ The ‘no-reflow' phenomenon is a concept that has been documented in clinical cases and animal models of cerebral ischemia triggered by occlusions as short as 30 minutes.^11, 12, 13^ Significant alterations in microvessel integrity occur within the first few minutes of flow cessation after an ischemic insult.^14^ These changes culminate in focal ‘no-reflow' within the microvessels, where the activation of endothelium and coagulation factors leads to the recruitment of leukocytes and platelets, obstructing the microvessel lumen and leading to an increase in microvessel permeability.^14, 15, 16^ The penumbral region borders the infarct core and contains stressed, but functionally viable, neurons. Timely reperfusion can salvage stressed neurons within the penumbral region, but if not achieved, continued hypoperfusion leads to the extension of the infarct core, which eventually envelops the penumbral region.^17, 18^

Two-dimensional optical imaging spectroscopy (2D-OIS) is a powerful imaging technique that uses different wavelengths of illumination to record image data from which hemodynamic parameters can be determined, including changes in HbO_2_ concentration at the cortical surface.^19^ Traditionally, studies using 2D-OIS investigate changes in the hemodynamic parameters between periods of cortical stimulation and rest. Here, we calculate the changes between different time points of the ischemic event.

Focal ischemia can be replicated in rodent models by middle cerebral artery occlusion (MCAo) whereby a siliconized filament is placed at the base of the MCA branch to cause occlusion.^20, 21^ Although many other forms of occlusion exist (coagulation of the artery or surgical ligature) such techniques are less controllable with respect to reperfusion after a limited period of occlusion than the filament MCAo method.^22, 23^ However, one drawback with filament MCAo is the larger intersubject variability with regard to the final infarct volume. In this study, we developed a remote filament method, whereby the filament is placed in the artery without blocking blood flow, to allow baseline recordings to be made, and is then advanced in the imaging setup to cause the focal ischemic event, with subsequent retraction after the desired length of time. Using this approach we are the first group to report the μmolar change in the concentration of HbO_2_ from the baseline perfusion, during the stroke period, and after block removal (reperfusion) for each animal *in vivo*. This allows us to correlate the dynamical changes of HbO_2_ perfusion with specific markers that can delineate a timeline of events that lead to neuronal injury^6, 16^ and that have already been identified as contributing to the ‘no-reflow' phenomenon, i.e., platelets and neutrophils, as well as markers of blood brain barrier (BBB) disruption, glial activation, and inflammation.

We hypothesise that the lack of sustained reperfusion and not the initial decrease in HbO_2_ during occlusion governs the extent of pathophysiologic damage and that platelet aggregation has a key role in the cerebral ischemic no-reflow phenomenon through physical obstruction of the microvasculature.

## Materials and methods

### Remote Filaments

Sterile Ethicon 5-0 monofilament sutures (Ethicon, Bridgewater, NJ, USA) were cut to 5 cm lengths and then marked at 10 mm from the tip for subsequent coating. Clear 0.5 mm diameter i.v. catheter tubing (Portex, Kent, UK) was cut to 2 cm length and used to aid stability during insertion of the remote filament. The filament tip was dipped in ethanol to remove any dirt or grease and covered evenly over the 10-mm length with a mixture of Xantopren M Mucosa and Activator NF Optosil.^24^ Filament diameter was checked under a microscope using a *μ*m scale (diameter ranged from 0.21 to 0.23 mm). Filaments were then suspended tip down and left to air dry for 1 hour.

### Animals

All procedures were performed under appropriate Home Office Licence (UK), adhered to the Animals Scientific Procedures (1986) Act and were performed in accordance with ARRIVE guidelines. Experiments and the study were approved by the local ethics committee in the Faculty of Life Sciences of the University of Manchester, UK. Mice were kept at 21°C and 65% humidity with a regulated 12-hour light–dark cycle and with free access to food and water. Fifteen male C57/BL6 mice weighing between 30 and 36 g (age 18 to 25 weeks) were used in this study. Nine C57/BL6 male mice underwent 30 minutes occlusion with 6 hours reperfusion (MCAo group); three underwent sham (SHAM) surgery, where the filament was left within the internal carotid artery during surgery but not advanced; and three control (CTRL) mice underwent a tracheotomy only. Animals in the sham group showed no significant variability in the measures between subjects and with the control animals using an ANOVA comparison. Therefore in line with the 3 R's for the humane use of animals in scientific research we kept the sham and control group at *n*=3.

### Surgical Preparation

Anesthesia was induced with 4% isoflurane (Abbott, Berkshire, UK) in room air. Once the animals were unconscious with lack of pedal reflex, they were maintained under 2% isoflurane *via* a face mask for surgery and, once a tracheal cannula had been inserted, 1% to 1.5% isoflurane for the remainder of the experiment.

For MCAo, a midline skin incision across the throat was made to expose the left common carotid artery, and its bifurcation into internal and external branches. After cauterizing the superior thyroid artery branching from the external carotid artery (ECA), the common carotid artery was ligated with a 10x2 mm micro-serrefine (Fine Surgical Tools (FST), DE).^21^ The ECA was fully occluded by cauterization as distally from the bifurcation with the internal carotid artery as could be achieved and an ~0.23 mm hole was cut into the ECA proximally. A standard Doccol 0.21±0.02 mm width filament (6021PK10, Doccol Corporation, Sharon, MA, USA) was inserted and advanced 5 mm. The custom-made remote filament was levered into the hole in the wall of the ECA while the Doccol filament was simultaneously removed. The filament was then further advanced to the internal carotid artery to a point just before the MCA branch, but without occluding the MCA. The filament was secured in place with a surgical suture loop around the ECA that was tight enough to prevent bleeding but would allow the movement of the filament. A 2-cm length of transparent 0.5 mm diameter i.v. catheter tubing was filled with silicon oil (Sigma-Aldrich, Gillingham, UK) and positioned close to the surgical site, holding the remote filament in place. The tubing was then secured on to the sternocleidomastoid muscle.

For imaging experiments, animals were fixed in a stereotactic frame (Narishige, Tokyo, Japan) with ear bars, mouth bar and a dorsal head post to prevent movement. Animals were artificially ventilated at 1% to 1.5% isoflurane in room air *via* a Zoovent Jetsys ventilator (Universal Lung Ventilators Ltd, Milton Keynes, UK). Body temperature was maintained at 36.9±0.2°C *via* a heating blanket controlled with a rectal probe (Harvard Apparatus, Kent, UK), and the heart rate was monitored *via* ECG throughout the experiment.

A midline incision in the scalp was made and the skull on the left side exposed. A soft paraffin (Vaseline) ‘well' was constructed directly onto the skull and filled with 37°C saline. This well was then closed with a glass coverslip to create a stable imaging window over the somatosensory cortex, through which the intact and translucent skull could be observed.

### Two-Dimensional Optical Imaging Spectroscopy

Two-dimensional spectroscopic imaging data were collected through the imaging window over the intact skull using a high signal-to-noise charged coupled device (CCD) camera (Pantera 1M30, DALSA, Munich, Germany). The region of interest was illuminated sequentially by four different wavelengths of light (550±10 nm, 560 ±10 nm, 577 ±10 nm, and 700 ±10 nm) using a Lambda DG-4 high-speed filter changer (Sutter Instruments, Novato, CA, USA). Camera data collection was synchronized with the filter changer so that each image frame was recorded with one of the four different cortical illumination wavelengths in a sequential manner at a rate of 28 Hz.

A ceramic attenuator (PI Instruments, Bedford, UK) was attached to a single whisker on the right whisker pad to enable computer-controlled mechanical stimulation of the barrel cortex throughout the experiment.

A single imaging experiment consisted of a continuous recording of 30 trials. Each trial was 16 seconds long and contained a 4-second prestimulus period; 4 seconds of 8 Hz mechanical whisker stimulation and an 8-second recovery time. These 8-minute experiments were recorded before, during, and after MCAo or the time equivalents in the other experimental groups. Supplementary Figure 1 shows the timing of a single trial, experiment and the timeline for the whole procedure.

### Induction of Cerebral Ischemia

Once the CCD camera was in position, an imaging region of interest was chosen to include branches of the MCA and surrounding parenchyma within the left somatosensory cortex. Visualization of the activation of the barrel from whisker stimulation provided instant feedback on the correct positioning. Two sets of trials were recorded for baseline comparison, after which the filament was externally moved ~2 to 3 mm to induce occlusion of the MCA. Experiments were recorded consecutively, for a total of 30 minutes, during the occlusion phase. The remote filament was then retracted back 6 mm to allow full reperfusion of the MCA. Trial sets were then recorded every 30 minutes for 6 hours after reperfusion.

After 6 hours reperfusion, the animal was transcardially perfused with 0.9% saline solution containing 0.5% sodium nitrate followed by 2% paraformaldehyde solution. After fixing, the brain was removed, stored in 2% paraformaldehyde for 24 hours and submerged in a 30% sucrose solution for a further 24 hours.

### Tissue Processing

Coronal brain sections (30 *μ*m thick) were cut on a sledge microtome (Leica, Milton Keynes, UK) with freezing stage (Bright Instruments, Huntingdon, UK). Sections were stored in antifreeze solution (30% ethylene glycol and 20% glycerol (Sigma, Gillingham, UK) in phosphate-buffered saline (PBS)) at −20 °C, before histological staining.

### Immunohistochemistry

Brain sections were washed in PBS and incubated for 1 hour at room temperature with 2% normal donkey or rabbit serum (Vector Labs, Peterborough, UK) in PBS containing 0.3% Triton X-100 (Sigma) to block nonspecific binding. Sections were then incubated overnight with primary antibodies as follows: mouse monoclonal anti-CD41/integrin alpha 2b antibody (platelets) (1:500, Abcam, Cambridge, UK), rabbit anti-Iba1 (microglia) (1:1,000, Abcam), rabbit anti-laminin antibody (1:500, Abcam), and rabbit neutrophil serum anti-SJC (clone SJC4, 1:1,000). Sections were further incubated with secondary antibodies conjugated to Alexa 488 nm or Alexa 594 nm fluorochromes (1:500, Invitrogen, UK) for 2 hours at room temperature. A final wash was performed in PBS and slices were mounted on charged microscope slides. Prolong Gold antifade reagent (with or without DAPI; Invitrogen) was used as the mounting medium for glass coverslips. Images were captured using an Olympus widefield BX51 upright microscope featuring 4x/0.13 UPlanFLN, 10x/0.3 UPlanFLN, and 20x/0.5 UPlanFLN objectives. Images were captured using a Coolsnap EZ camera (Photometrics, Herts, UK) and MetaVue Software (Molecular Devices, Sunnyvale, CA, USA). Specific band-pass filter sets were used for DAPI (31000v2, BP350/50 nm), Alexa 488 (FITC 41001 BP480/40 nm), and Alexa 594 (Texas Red 41004 BP560/55 nm). Settings remained constant, and a selected area of the cerebral cortex at the same magnification (x20) was used across groups for each specific marker.

Premounted sections for IL-1*α* and IL-1*β* immunohistochemistry were performed to detect endogenous IL-1*α* and IL-1*β* expression. Sections were mounted onto charged slides before antigen-retrieval step, during which slides were submerged in citrate buffer (Invitrogen) diluted 1:100 in distilled water. Slides in buffer were heated in a microwave for 10 minutes to remove paraformaldehyde-induced crosslinks within the tissue. Sections were blocked with endogenous peroxidase consisting of 10% methanol (Fisher, Loughborough, UK) with 3% H_**2**_O_**2**_ (Sigma) in PBS containing 0.3% Triton X-100 (Sigma). Sections were washed in PBS and incubated for 1 hour at room temperature with 2% bovine serum albumin (Sigma) with 5% normal rabbit serum (Vector Labs) in PBS containing 0.3% Triton X-100 (Sigma) to block nonspecific binding, followed by overnight incubation at room temperature with primary goat anti-mouse IL-1*α* or IL-1*β* IgG antibody (1:500, R&D Systems, Minneapolis, MN, USA), and subsequent incubation with a biotinylated rabbit anti-goat antibody (1:500, Vector Labs) for 2 hours. Sections were incubated for 1 hour with Vectastain ABC kit (Elite Standard; Vector Labs) followed by nickel ammonia diaminobenzidine (DAB) staining—with a 0.1-mol/L sodium acetate wash to initiate and stop DAB reaction—followed by a final dehydration step, in which slices were incubated in ascending ethanol concentrations (70%, 90% and 100%) for 5 minutes each, followed by a further 5-minute incubation in Ultraclear (TAAB Laboratories, Berks, UK). Slides then underwent counterstain with cresyl violet (CV; see protocol below) before coverslipping with DPX mounting medium (Agar Scientific, Stansted, UK). Full-slice images were captured using a brightfield Olympus S2X9 microscope with GXCAM-1.3 video capture and highlight 3100 power source. GX-capture software was used for image acquisition (Camera and software from GX Optical, Stansfield, UK). Magnification and light-illumination settings remained constant for capture of all images.

Extravasation of IgG through the leaky BBB and into the ipsilateral hemisphere was detected by mouse IgG antibody DAB staining as described above. IgG is easily detectable as it is found in large quantities within systemic plasma (550 to 1900 mg/dL).^25^ The presence of IgG within the brain parenchyma is therefore a reliable measure of BBB disruption.^26^ Free-floating immunohistochemistry was performed to detect the leakage of endogenous IgG into the brain parenchyma as a measure of BBB breakdown. Slices were loaded into wells with PBS and endogenous peroxidases blocked with 1% H_2_O_2_. A second blocking agent, consisting of 5% normal horse serum (Vector Labs) and 2% bovine serum albumin in PBS containing 0.3% Triton-X-100 (Sigma), was applied for 1 hour. Sections were incubated with biotinylated horse anti-mouse IgG antibody (1:500, Vector Labs). Sections were incubated for 1 hour with ABC kit followed by DAB staining with 0.1 mol/L sodium acetate wash to initiate and stop DAB reaction. Sections were mounted on charged slides, dried and coverslipped using DPX mounting medium.

### Cresyl Violet Staining

Brain sections were mounted on gelatinized slides and initially soaked in alcohol of increasing concentrations 50%, 70%, 90%, and 100% followed by descending order again (4 minutes each concentration) to remove lipids and fixation chemicals from the tissue, sections are then submerged with 1.5% CV staining solution. Sections underwent ascending alcohol concentrations 50%, 70%, 90% and 100% (2 minutes each concentration) to dehydrate the tissue. Ultraclear (4 minutes) was used as a clearing agent, making unstained parts of the tissue transparent followed by coverslipping with DPX mounting medium.

### Data Analysis

#### Immunohistochemistry

Images of the cerebral cortex were collected from five evenly spaced sections spanning the somatosensory cortex from both hemispheres for comparison. IL-1*α* and IgG were quantified using changes in measured pixel density between the cortex in the stroked left hemisphere and the corresponding right hemispheres, using Image J software (National Institute of Health, USA). IgG and IL-1*α* were quantified by measuring the mean gray value (pixel density) of each hemisphere, the value of which was subtracted from the absolute white value (max), and comparing hemispheric values. Activated microglia (Iba-1) and platelet cells (CD41) were double-blinded to eliminate bias and then counted manually and in each hemisphere in a 0.46 × 0.34 mm size region of the somatosensory cortex. Cresyl violet-positive (CV) cells for cortex counts were centred on layer IV with a 0.3 × 0.3 mm region of interest for all animals.

#### Spectroscopic imaging

**Figure 1:**
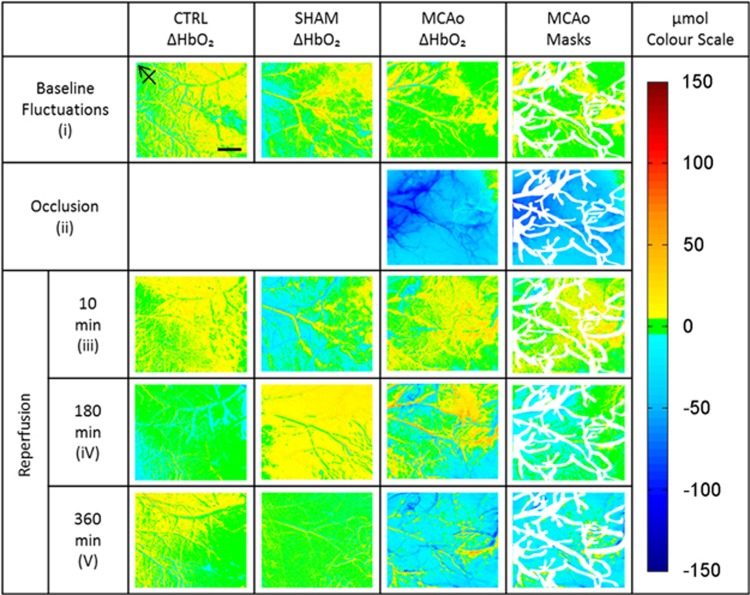
Example color maps of ΔHbO_2_ in the three experimental groups. The first two columns show the maps over the time course of the experiment for the control and sham group. For the total time of nearly 7 hours animals in both groups are very stable and show only small variations in the HbO_2_ concentrations. The third column of maps shows a typical animal in the middle cerebral artery (MCA) occlusion (MCAo) group. The sudden decrease in the HbO_2_ concentration gives instant feedback that the occlusion was successful. When removing the filament we can see increased HbO_2_ in the branches of the MCA coming in from the left and the microvasculature supplying the parenchyma. This initial reperfusion then fades away over time. The masked maps on the right show the areas that were considered for further analysis. Maps in the first two rows show change with respect to a time point before occlusion and the reperfusion maps show change with respect to the end of the occlusion (scale bar =1 mm, arrow points rostral).

First, imaging frames were separated according to illumination wavelength, resulting in an effective frame rate of 7 Hz. These single-wavelength data stacks were then averaged to obtain one single grayscale image per wavelength for each set of 30 trials. The dependency of multiwavelength optical imaging on photon pathlength was corrected for using a modified Beer-Lambert Law.^19, 27^ In the conversion from grayscale to attenuation the sets of four images were compared between different time points within the experiment. For example, to establish the baseline variability in cortical HbO_2_ levels, we compared the two image sets recorded before occlusion. To establish the extent of the decrease in HbO_2_ during occlusion, we compared the set before occlusion with those at the end of the 30-minute occlusion. All the remaining oxygenation maps for the MCAo group were calculated by comparing the data at the end of occlusion against the maps recorded in the 6 hours after the filament was withdrawn (see Figure 1, third column of maps). For the other two experimental groups (sham and control), we compared the equivalent time points, though no actual occlusion took place (Figure 1, first and second column of maps). Once these maps were created, we produced a single mask for each animal to exclude the large arteries and veins from further analysis (Figure 1, fourth column of maps). This approach allowed the calculation of *μ*molar changes in HbO_2_ concentration in the area of microvascular perfusion of the parenchyma before, during and after occlusion, as well as comparisons between groups.

For the visualization of the ΔHbO_2_ we created a *μ*mol color scale, with blue for a decrease, red an increase and with green colors indicating little change compared with the reference time point (Figure 1, far right). For statistical evaluation, the mean pixel values of the regions outside the masks of the ΔHbO_2_ maps were calculated. Due to a slight variability of carrying out the complex experiment, the timing within the MCAo group for the data acquisition during early reperfusion ranged from 8 to 15 minutes after block removal and later reperfusion ranged from 345 to 360 minutes. The timing of baseline recordings and recordings during occlusion was precise up to the minute.

### Statistical Analysis

Animals were randomized for experiments and quantitative analyses were double-blinded across all groups; control group *n*=3, sham group *n*=3, MCAo group *n*=9 (one mouse was excluded from group due to hemorrhage). One-way ANOVA was used to compare mean HbO_2_ concentration change in *μ*molar values outside the masked regions in the imaging data, coupled with Bonferroni multiple comparisons test in histology data to indicate differences between the ipsilateral and contralateral hemispheres, calculated and displayed as mean values with error (±) displayed by standard deviation. A Pearson's rank correlation coefficient test was performed to measure the strength of association between mean changes in ΔHbO_2_ concentration during occlusion and during late reperfusion with CD41, Iba-1, IgG, IL-1*α*, and CV data.

## Results

### Imaging of Loss in Reperfusion After Middle Cerebral Artery Occlusion

**Figure 2:**
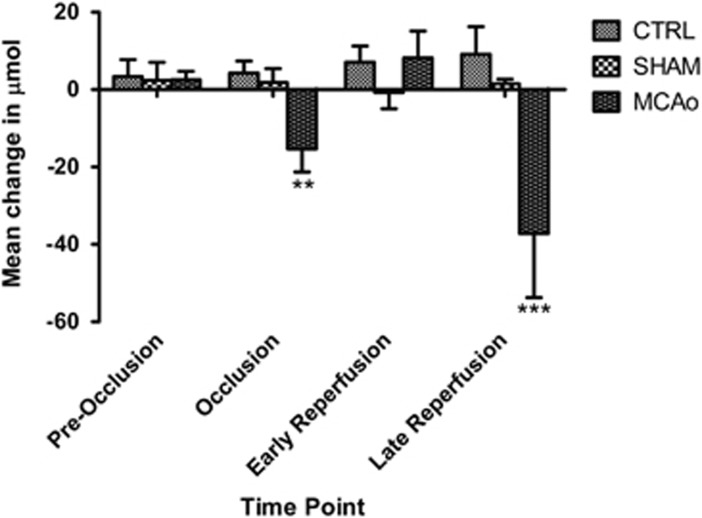
Changes in oxyhemoglobin concentrations. Variations in ΔHbO_2_ for all three groups at the preocclusion time point are small and stable. The middle cerebral artery occlusion (MCAo) group shows a significant decrease in HbO_2_ during occlusion. The other two groups remain stable at the equivalent time points. All of the following changes are calculated with respect to the occlusion value. There is an initial increase at the early reperfusion time point in the HbO_2_ concentration for the MCAo group after removing the filament. But by the late reperfusion time point the HbO_2_ concentration for the MCAo group has decreased significantly below the concentrations at the end of occlusion. The other two groups show some fluctuations but the changes are not significant.

Before investigating the change in HbO_2_ concentration caused by ischemia, we established the interexperiment variability of the HbO_2_ parameter (without any manipulation of blood flow using the filament). Baseline recordings from the three experimental groups show small fluctuations of ΔHbO_2_ between trials of 2.55±2.20 *μ*mol in the MCAo group, 2.37±4.7 *μ*mol for the sham group and 3.4±4.36 *μ*mol for the control group (see Figure 2, Preocclusion). Nonsignificant variation was observed between mean [HbO_2_] values for first baseline readings compared with second baseline readings across the experimental.

Once we moved the remote filament forward in the MCAo group the ΔHbO_2_ displayed an 8-fold decrease within the stroked hemisphere. ΔHbO_2_ values decreased to −15.36±5.93 *μ*mol compared with baseline level. Over the same time period, the fluctuations in the sham and control group remained at baseline level with ΔHbO_2_ sham 1.85±3.57 *μ*mol and control 4.31±3.1 *μ*mol (Figure 2, Occlusion).

After 30 minutes of occlusion, we retracted the filament to allow reperfusion to occur. There is an instant 2-fold increase in oxygenated blood supply within microvessels supplying the brain parenchyma with ΔHbO_2_ values of 8.22±6.89 *μ*mol compared with levels at the end of the occlusion period (Figure 2, Early Reperfusion). Again, in the sham and control groups, which did not undergo occlusion, the HbO_2_ concentration recorded at the equivalent time points remains similar to baseline fluctuation levels with ΔHbO_2_ values for sham −0.80±4.12 μmol and control 7.11±4.13 μmol.

Over the 6 hours after retracting the remote filament occluding the MCA we saw a significant decline of the ΔHbO_2_ concentration. The decrease of ΔHbO_2_ in the MCAo group is −37.23±16.52 *μ*mol compared with the last recording during occlusion. End point sham and control animal ΔHbO_2_ were recorded at 1.54±1.14 *μ*mol and 9.15±7.11 *μ*mol, respectively (Figure 2, Late Reperfusion).

### Significant Change in Biomarkers of Ischemic Injury 6 Hours After Stroke

**Figure 3:**
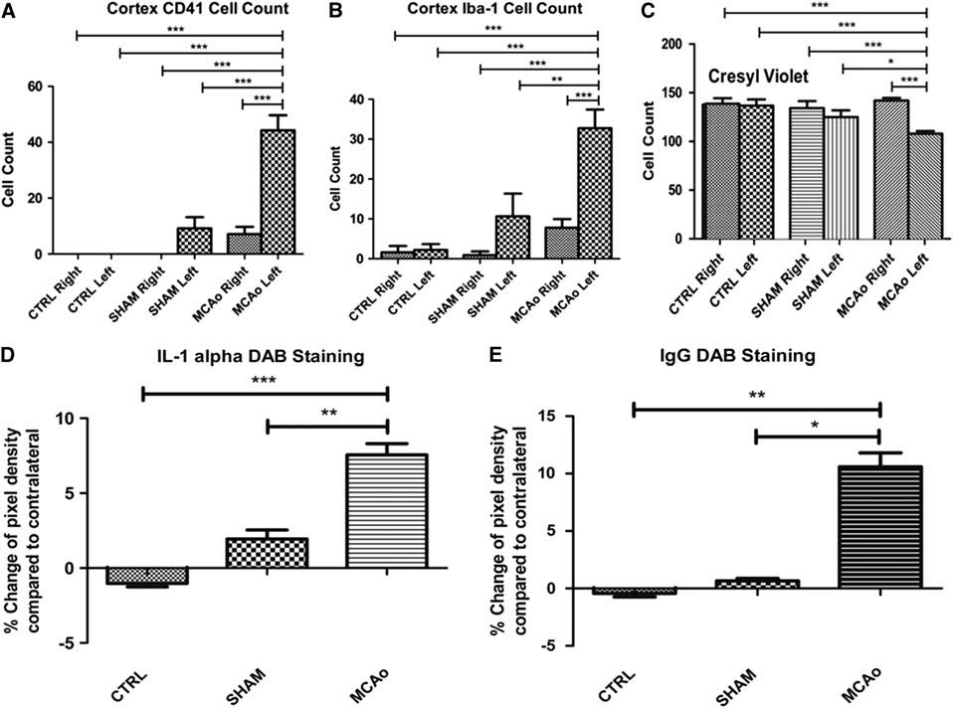
Significant increase in biomarkers for acute ischemic injury and inflammation 6 hours after middle cerebral artery occlusion (MCAo). (**A**–**C**) One-way ANOVA with *post hoc* Bonferroni multiple comparisons test to compare cell counts within the MCAo group's stroked left cortex to their right cortex and other group hemispheres. (**D**, **E**) One-way ANOVA with *post hoc* Bonferroni multiple comparisons test to compare pixel density of DAB between the two hemispheres in all three groups. CTRL group *n*=3, SHAM group *n*=3, MCAo group *n*=9.

Known markers of acute ischemic injury like CD41+ showing platelets, Iba-1+ staining microglia, IgG and IL-1*α* as markers of inflammation and BBB breakdown, differed significantly between the cortex of the stroked left hemisphere of the MCAo group mice compared with their right cortex (see Figure 3 and Supplementary Figure 2). These changes were also significant compared with the cortices in either hemisphere of the sham and control group. These data provide further evidence that an ischemic challenge was initiated using the remote filament and a 30minute MCAo.

In the stroked animals, the 30-minute MCAO induced a 44% increase in numbers of CD41+ platelets (*P*<0.01) in the left cortex compared with the left cortex of the control group and a 40% increase (*P*<0.01) compared with sham group (Figure 3A). The MCAo also induced a 31% increase in activation of Iba-1+ microglia (*P*<0.01) in the stroked cortex compared with control equivalent and a 22% increase compared with sham group (Figure 3B). Detection of the pro-inflammatory cytokine IL-1*α* in the stroked hemisphere was up by 7.55% compared with an increase of 1.95% in the sham animals and −1.02% in the control group (Figure 3D; *P*<0.01). Leakage of systemic protein IgG into the stroked brain hemisphere was detected as an increase of 10.62% compared with the right hemisphere (Figure 3E). This difference between the two hemispheres was significantly bigger than the difference in the sham (0.66%) and control group (−0.42% *P*<0.01). Cresyl violet stain revealed a reduction as well as morphological changes of the cortical neurons in the stroked hemisphere of the MCAo group, indicative of dying cells. Neurons were more condensed with smaller cell bodies and displayed a 24% (*P*<0.01) reduction in CV-stained cells compared with other hemisphere as well as 18% 20%, 21%, and 22% compared with sham ipsilateral and contralateral, control ipsilateral, and contralateral hemispheres, respectively (Figure 3C). Supplementary Figure 2 A1 and A2 displays the CV stains from an MCAo animal.

### Late Reperfusion Deficit Correlates with Increased Pathology

**Figure 4:**
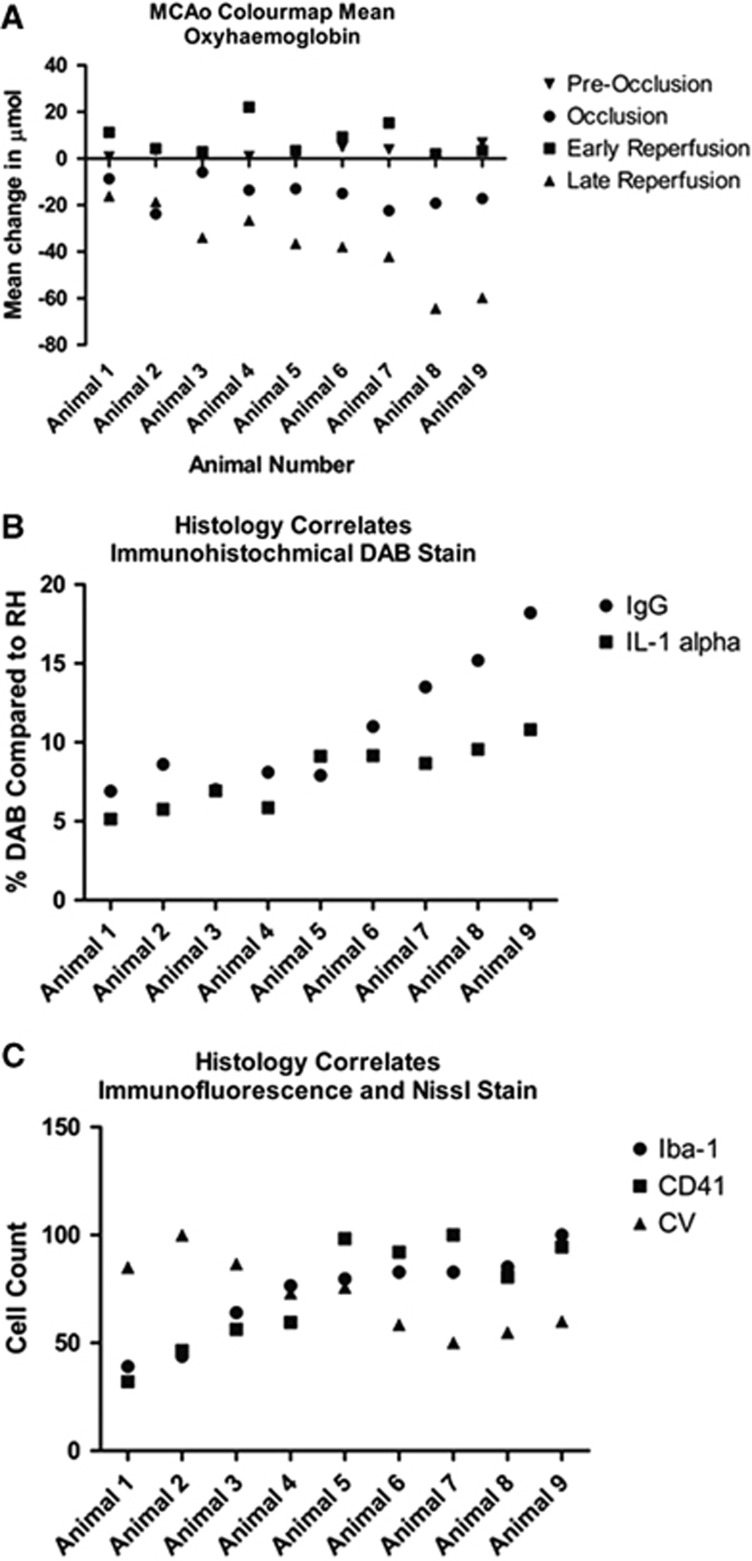
ΔHbO_2_ during late reperfusion correlates with histological biomarkers. (**A**) Animals 1 through 9 were ranked according to their ΔHbO_2_ value at 6 hours after reperfusion. (**B**) End-point ΔHbO_2_ correlates with increases in blood brain barrier (BBB) breakdown (represented by higher IgG) and pro-inflammatory mediator IL-1*α*. (**C**) Immunofluorescent staining of Iba-1-positive microglia and CD41-positive platelet cells reveals a correlation represented by a monotone increasing curve. As expected cresyl violet (CV) stain displays a decreasing curve.

To establish whether it is the lack of oxygenation during the acute phase of the MCAo or the later decline in HbO_2_ concentration that contributes most to the pathological damage shown in the paragraph above, we ranked the individual animals in the MCAo group according to the magnitude of ΔHbO_2_ at the late reperfusion time point (Figure 4A). Using this ranking we then plotted the magnitude of the histological correlates of the DAB stains for IgG and IL-1*α* (Figure 4B) and the immunofluorescence stains and Nissl stain (Figure 4C). All the plots display a monotone relationship between the late reperfusion deficit and the histological biomarkers.

To quantify this correlation, we performed a Pearson's rank coefficient test to measure the strength of association between the mean change in HbO_2_ during occlusion and late reperfusion with known histological markers of acute ischemic injury (Iba-1, CD41, IgG, IL-1*α* and neuronal cell count) (see Table 1). The Pearson's rank coefficient revealed that there is no significant correlation in the change of HbO_2_ concentration during the acute ischemic event and the histological biomarkers in the MCAo group but there is a significant correlation for the late reperfusion time point. The Pearson's rank coefficient found a significant association between late reperfusion ΔHbO_2_ values and Iba-1 (*P*<0.01), CD41 (*P*<0.05), IgG (*P*<0.01), IL-1*α* (*P*<0.01), and neuronal cell count (*P*<0.05).

## Discussion

By using a remote filament design in combination with 2D-OIS, we are the first group to quantify in the same animal the changes in HbO_2_ concentration before, during, and after ischemia in a preclinical model of stroke with high spatial and temporal resolution. Our findings show that it is not the initial decrease in HbO_2_ during occlusion that correlates to the histological biomarkers but the ‘no reflow' event, after block removal and subsequent reperfusion that predicts pathophysiologic outcome. Thus, the level of inflammation with neuronal damage correlates with secondary decline of HbO_2_ and not occlusion.

Our study shows that the design of a remote filament allows recordings of baseline HbO_2_ to be obtained and compared with occlusion and reperfusion states. The observed decrease in HbO_2_ during occlusion fits with the previous findings^28, 29^ where similar changes in HbO_2_ were sufficient to induce neuronal damage.^28^ This response reveals the presence of an ischemic stroke; HbO_2_ levels decrease due to the cessation of blood flow to the region of interest (left somatosensory cortex). The lack of a resupply of oxygenated blood coupled with the continued respiration of neurons results in dramatic decreases in HbO_2_ levels in the stroked cortex.^30, 31^ The lack of significant change in sham and control data throughout the experiment reveals that HbO_2_ changes observed within the MCAo group result from the transient filament occlusion and not overall decline of the animal through the course of the experiment.

Upon reperfusion, HbO_2_ levels increase within the major vessels and parenchymal regions, coinciding with the hyperemia phase that has been previously documented.^32, 33^ Hyperemia occurs as flow is restored to the cerebral cortex after removal of the block, reintroducing oxygenated blood. This hyperemia upon reperfusion is thought to arise from vasoparalysis: loss of autoregulation of cerebral blood flow due to accumulation of lactic acid and vasoactive mediators during occlusion.^32, 33^ Over time, reperfusion dissipates: the concentration of HbO_2_ starts to decrease within the parenchyma, with only the major vessels retaining increased HbO_2_ levels as shown in Figure 1 similar to events reported by Kuo *et al.*^28^ This dissipation could be linked to the presence of CD41 positive platelets that stick to the microvessel walls because of adhesion molecule upregulation on the surface of endothelial cells forming micro-thrombi aggregates within the stroked hemisphere (Figure 3A). Ischemia-induced hypoxia is a powerful stimulus for coagulation initiation and can thus lead to deficits in reperfusion.^14, 15, 34^ Hypoxia-induced platelet accumulation within the microvasculature contributes to the ‘no reflow' phenomenon.^12, 14, 35^ A state of no-reflow is established around 5 hours after reperfusion, leading to neuronal loss. There appears to be an overflow effect occurring within the contralateral hemisphere of the MCAo group whereby increased numbers of CD41-positive platelets and Iba1-positive activated microglia were found. This suggests that a dampened pathophysiologic cascade has been initiated within the contralateral (non-lesioned) hemisphere as well as the ipsilateral (lesioned) hemisphere after 30 minutes of focal ischemia.

Known markers of early inflammatory processes, microglia activation, BBB breakdown, and neuronal loss were observed after the first 6 hours of reperfusion. Pathophysiologic changes at this early time point are not well understood and many studies have focused on time periods of more than 12 hours after stroke.^8, 36^ Furthermore, very few studies in mice have used a transient model of MCAo to investigate the acute phase of reperfusion. Neuronal injury triggers activation of the resident immune cells of the brain, microglia, upregulating Iba-1 protein at cell surface and initiating early inflammatory processes.^7, 37^ Activation of microglia leads to the production and release of proinflammatory cytokines, including IL1*α* within 8 hours of reperfusion, which then switches to IL-1*β* after 8 hours.^9^ IL-1*α* induces vascular endothelial cell expression of the adhesion molecules ICAM-1 and VCAM-1.^38^ These adhesion molecules trap platelets and circulating leukocytes in the lumen of microvessels, which contribute to BBB breakdown. Evidence of BBB disruption was indicated by the presence of IgG protein in the brain parenchyma (Figure 3E). IgG is present within the plasma of the circulation throughout the body, but does not pass the BBB unless disruption has occurred.^8, 25, 26^ Our results show neuronal loss by 6 hours after reperfusion, supporting findings recorded from permanent occlusion studies in rats.^36, 39, 40^

It is widely believed that the main predictor for pathophysiologic outcome and neuronal injury occurs as a consequence of stroke severity whereby the worse the stroke, the worse the outcome. We show however that the HbO_2_ decrease during occlusion does not correlate with pathophysiologic outcome, but that it is the HbO_2_ value at the end of 6 hours reperfusion that is associated with pathophysiologic outcome.

In this study, we compared individual animals mean ΔHbO_2_ at occlusion and at late reperfusion with immunohistochemistry markers. The test revealed a strong correlation between the mean change in *μ*mol of HbO_2_ values at late reperfusion with increased levels of pathophysiologic markers—CD41+ platelet aggregates, Iba-1-positive activated microglia, IgG (representative of BBB breakdown), IL-1*α* expression—as well as decreased numbers of CV-stained neurons. A flow-dependent response governs ischemic pathology, as lack of reperfusion at acute time points correlates with decreased neuronal survival.

Platelet aggregates present within the microvasculature of the ischemic cerebral cortex were significantly more prevalent in mice where the lack of reperfusion at late stages was most severe. This reveals a strong correlation between the outcome of reperfusion and the number of platelet aggregates, suggesting an association between increased numbers of platelet and lack of sustained reperfusion within the cerebral cortex. Platelets produce and release the proinflammatory cytokine IL-1*α*, which was detected in the stroked hemisphere after 6 hours of reperfusion.^38^ This was also seen in our MCAo animals.

We have demonstrated that 2D-OIS is an excellent tool to investigate the spatial and temporal dynamics of HbO_2_ changes before, during, and after stroke. One disadvantage of these optical approaches is that data acquisition is limited to cortical areas at the surface of the brain. This is less of a problem in the lissencephalic brain of a mouse but could be problematic in species with a more folded cortex. Furthermore, it would be interesting in the future to measure the actual tissue oxygenation with either Clark style electrodes or oxygen-sensitive MRI.

In conclusion, we show for the first time a method whereby an ischemic stroke can be fully controlled within a mouse model in such a way to allow the acquisition of baseline images for comparison with occlusion and reperfusion states in the same animal. Spectroscopic analysis of the imaging data produces color maps that represent *μ*molar changes in the concentration of HbO_2_, which show the effect of an ischemic challenge on hemodynamic responses with high spatial and temporal resolution. Immunohistochemistry analysis confirms that the ischemic challenge triggers early inflammatory biomarkers of ischemia and inflammation. The lack of reperfusion at later time points correlates with decreased neuronal survival, suggesting a flow-dependent response. There is no correlation with the acute decrease in HbO_2_ concentration during the occlusion. This finding underpins the importance for future stroke research to focus on overcoming the mechanisms that lead to a reduction in late HbO_2_ perfusion. The ability to gain baseline recordings for comparison within the same animal can create a platform for drug development and testing as well as comparison between comorbidity groups.

## Figures and Tables

**Table 1:** Correlation between ΔHbO_2_ and pathophysiology

		*Iba-1*	*CD41*	*IgG*	*IL-1α*	*Neuronal cell count*
Occlusion	Pearson corr	−0.2119 (ns)	0.3514 (ns)	−0.5402 (ns)	−0.3063 (ns)	0.2938 (ns)
ΔHbO_2_	Sig. (2-tailed)	0.5842	0.3538	0.1332	0.4227	0.4428
	*N*	9	9	9	9	9
Late reperfusion	Pearson corr	−0.8459**	−0.7111*	−0.8693**	−0.9170***	0.7672*
ΔHbO_2_	Sig. (2-tailed)	0.0041	0.0317	0.0023	0.0005	0.0158
	*N*	9	9	9	9	9

A Pearson's correlation coefficient test reveals a correlation between pathophysiologic biomarkers and experimental end-point mean ΔHbO_2_ values. Total of nine pairs analyzed per animal for each marker.
