# Supplementary material for: Delayed reperfusion deficits after experimental stroke account for increased pathophysiology
Source: J Cereb Blood Flow Metab. 2014 Nov 19;35(2):277–84. doi: 10.1038/jcbfm.2014.197 (PMC4426745; doi:10.1038/jcbfm.2014.197)
Supplement: Supplementary Figures [file jcbfm2014197x1.doc]

**
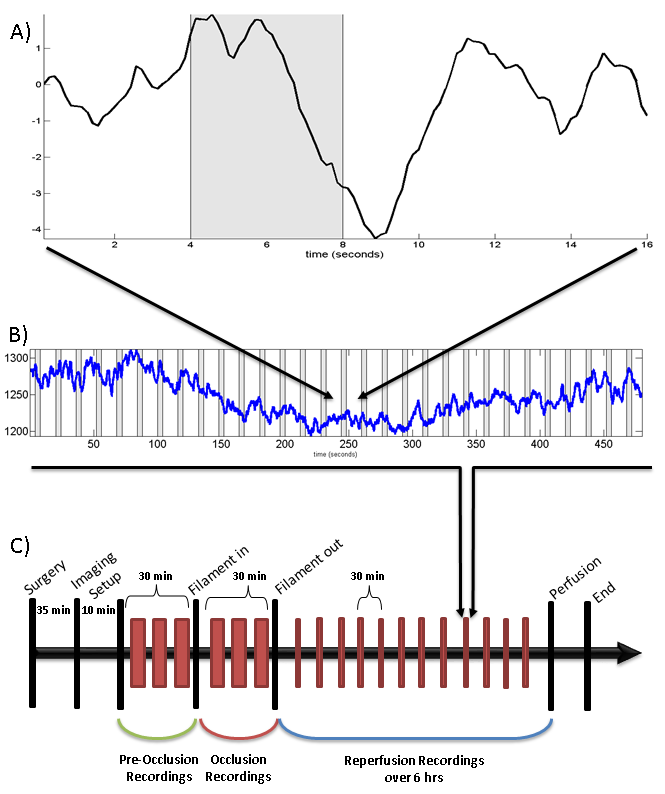
**

**Supplementary figure 1: A)** shows the time course for the change in mean grey value of the imaged ROI for one wavelength during a single trial. A single trial consists of a 4s pre-stimulus period. Then a single whisker gets mechanically stimulated for 4s (grey background). Then we record for another 8s to allow the signal to return to baseline before the next trial starts. **B)** shows the absolute grey value over 8 minutes in which we repeat the trial 30 times. Stimulation periods are again shown by the grey background. **C)** shows the timeline of the whole experimental procedure. The red bars show the time points during which imaging data are recorded as shown in B). Note that the timeline is representative and not to scale.


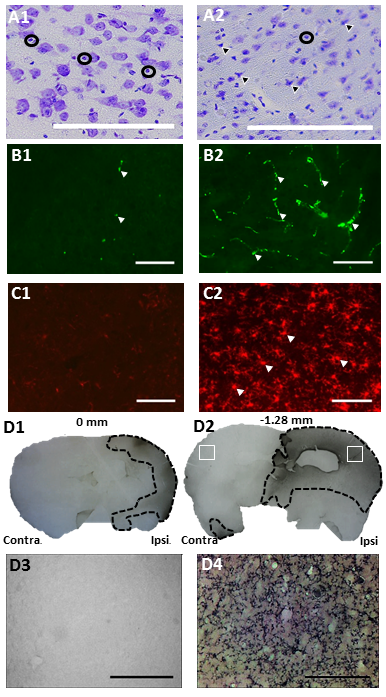


**Supplementary figure 2:** **Histology of pathophysiological markers of ischaemic stroke.** The right column shows the stoked hemisphere and the left column the corresponding contralateral hemisphere in the same slice except D1 and D2 that show whole coronal sections. **A1) and A2)** 40 times magnification of contralateral and ipsilateral cortical MCAo group CV stained neurons. Arrows in the ipsilateral hemisphere point towards increased numbers of cells which display a shrunken and pyknotic morphology, indicative of cell death. Circles highlight a number of CV stained glial cells (small and spherical in morphology). **B1)** and **B2)** show the staining for CD41+ platelet aggregates. The contralateral hemisphere shows little positive stain. In the image of the stroked hemisphere arrows point to a number of CD41+ platelet aggregates. **C1)** and **C2)** Contralateral and ipsilateral staining for Iba-1+ microglia. The contralateral hemisphere shows no positive stain. In the image of the stroked hemisphere arrows point to a number of Iba-1+ activated microglia. **D) BBB breakdown was significantly increased in the stroked hemisphere of MCAo mice.** Images are coronal sections from the brains of C57/BL6 mice. **D1)** and **D2)** display whole coronal slices at different stereotaxic coordinates in the brain of a typical MCAo group mouse. IgG detection is shown to be localized mainly to the ipsilateral hemisphere. Dotted line demarcates most intense DAB stained regions. **D3)** and **D4)** show the slice in D2) at higher magnification. The square ROI’s in D2 show the location of the images displayed in D3) and D4). All scale bars display 100 μm in length.
